# Supplementary material for: Aphid infestation differently affects the defences of nitrate-fed and nitrogen-fixing Medicago truncatula and alters symbiotic nitrogen fixation
Source: Proc Biol Sci. 2020 Sep 2;287(1934):20201493. doi: 10.1098/rspb.2020.1493 (PMC7542793; doi:10.1098/rspb.2020.1493)
Supplement: Figure S1;Figure S2;Figure S3;Supplementary file 1: Primer sequences and statistical data;Supplementary file 1: Primer sequences and statistical data [file rspb20201493supp1.pdf]

Supplementary Electronic Materials

PANDHARIKAR Gaurav, GATTI Jean-Luc, SIMON Jean-Christophe, FRENDO Pierre, POIRIE Marylène

Aphid infestation differently affects the defences of nitrate-fed and nitrogen-fixing *Medicago truncatula* and alters symbiotic nitrogen fixation.

DOI: RSPB-2020-XXXX

Supplementary Figures

Figure S1. Experimental design, plants and aphids materials.

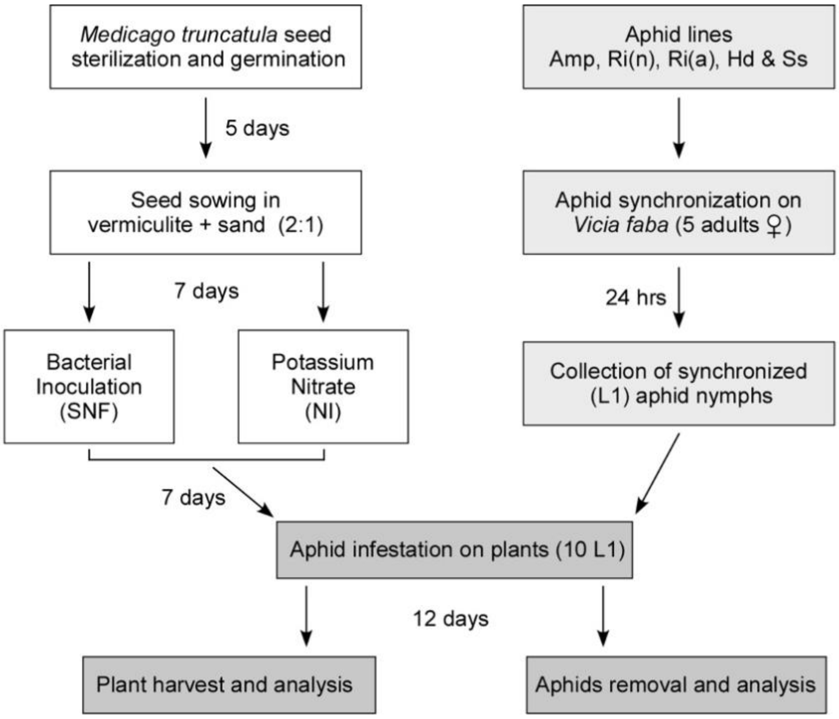

Plants. *Medicago truncatula* Jemalong A17 seeds were scarified for 3 min with 10% (v/v) commercial bleach and washed five times with sterile water before being put on 0.4% agar plates in the dark for 2 days at 4°C and then for 2 days at 20°C. After germination, 6 seedlings were transferred to a round pot (diameter x height: 7.5 x 7.5 cm) containing a mixture of vermiculite and sand (2:1) and all the pots were moved to a growth chamber at 23°C (16h light) and 20°C (8h dark), 60 ± 5 % relative humidity and watered with nitrogen-free Fahraeus medium. One week after the transfer, the pots were separated in two groups: one was inoculated with *Sinorhizobium meliloti* 2011 strain (SNF plants), the other was supplemented with 10 mL of 5 mM potassium nitrate (KNO_3) water, (NI plants). For inoculation, *S. meliloti* 2011, resistant to streptomycin, was cultured on Luria-Bertani medium with 2.5mM $CaCl_2$ and $MgSO_4$ and 200 µg/mL streptomycin for 3 days at 30°C. Subsequently, bacteria were grown in LBMC liquid medium for 24hrs, bacterial cells were pelleted at 5000g, washed twice with sterile distilled water and resuspended in sterile distilled water to a final optical density of 0.05 (OD 600 nm). Each SNF pot was supplemented with 10 mL of *S. meliloti* suspension.

Aphids. To obtain synchronized L1 aphids for infestation, five apterous adult females of each line were placed on separated fava bean leaves 24h before infestation and allowed to give birth to young nymphs. L1 nymphs were then collected and transferred to plants. After aphid infestation, the pots were isolated individually in an aerated plastic box and maintained at 20°C under a 16:8h light/dark photoperiod, with a 70% relative humidity. The aphids were kept on the plants for 12 days after the beginning of the aphid infestation (before aphids reached their adult reproductive phase).

Figure S2. Survival of pea aphids from the different lines on *M. truncatula* SNF and NI plants.

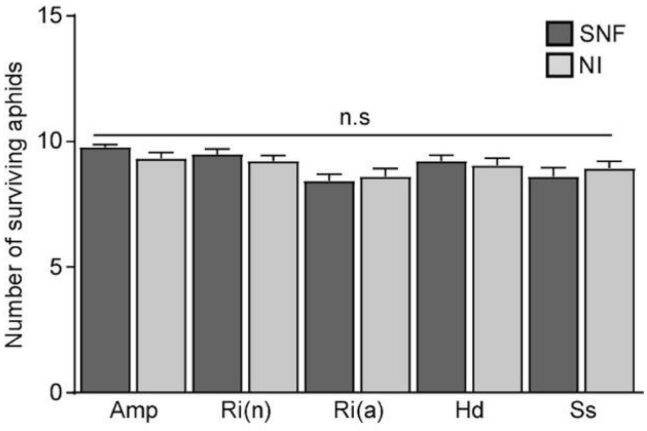

Figure S2: Survival of pea aphids from the different lines on *M. truncatula* SNF and NI plants. Mean number of adult aphids surviving after 12 days on inoculated and non-inoculated plants; no significant statistical difference was observed between the different YR2 lines and the two nutritional conditions of the plant (Mean ± s.e.; n= 4).

Figure S3. Comparison of the fresh and dry weight of SNF and NI plants before aphid infestation.

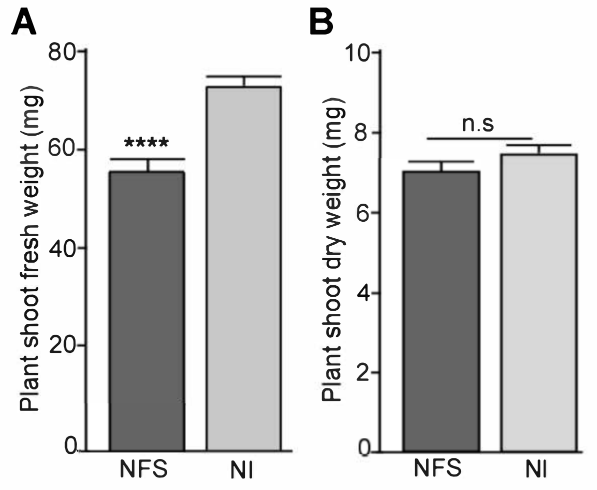

Figure S3: Comparison of the dry and fresh weight of NFS and NI plants before aphid infestation. A) Fresh weight and B) Dry weight of 2-week-old NFS and NI plants just before aphid infestation. Mean ± SE n=4. t-test: n.s., non-significant $p > 0.05$; **** $P \leq 0.0001$

Figure S4. Relative level of expression of *PR1* and *PI* genes in SNF or NI control plants.

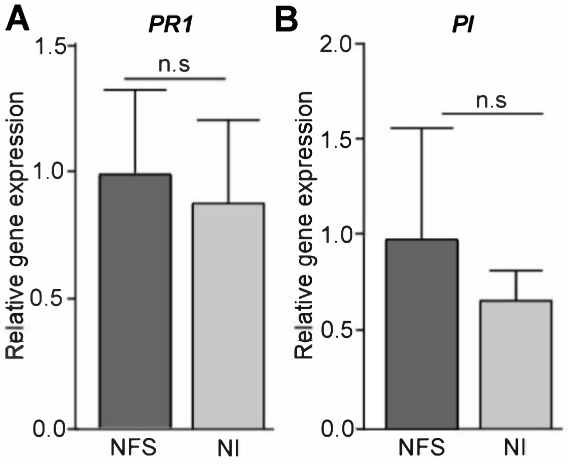

Figure S4: Relative gene expression of A) *PR1* and B) *PI* genes in NFS or NI control plants. *PR 1* and *PI* gene expression were set to 1 for NFS plant shoots and rescaled accordingly for NI plant shoots. Mean ± SE n=4. t-test:n.s., Non-significant.

Figure S5. Relative gene expression of the *PI* gene in the roots of NFS plants.

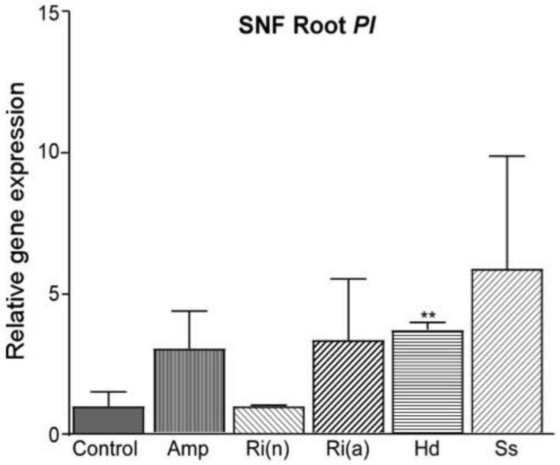

Figure S5: Relative gene expression of the PI gene in the roots of NFS plants. PI gene expression were analyzed in SNF plant roots as described for plant shoots. Expression of the control was set to one and rescaled accordingly for the aphid conditions. (Mean± SE, n=4). n.s., non-significant;** P ≤ 0,01

Supplementary file 1: Primer sequences and statistical data

Table S1. Primer sequences and conditions for qPCR analysis.

Real-time qPCR was performed as follows (AriaMx Real-time PCR machine, Agilent): 95°C for 3 min followed by 40 cycles at 95°C for 5 sec and 60°C for 30 sec. The primers efficiency was evaluated on a slope of a standard curve generated using a serial dilution of the samples. Cycle threshold values (Ct) were normalized to the average Ct of two housekeeping genes *Medtr2g436620* also named *MtC27* (the homolog of *M. sativa* translationally controlled tumour protein Msc27), and *Medtr4g109650.1* also named *a38* coding for a hypothetical protein. The expression of these two genes was not affected by the treatments in our experiments. The original Ct values were obtained from the machine software (Ariamix software; Agilent).

Description	Name	Forward primer	Reverse primer	Genomic ID	References
Housekeeping gene	*Mtc27*	TGAGGGAGCAACCAAATACC	GCGAAAACCAAGCTACCATC	Medtr2g436620	Del Guidice et al., 2011 [37]
Housekeeping gene	*a38*	TCGTGGTGGTGGTTATCAAA	TTCAGACCTTCCCATTGACA	Medtr4g109650	Del Guidice et al., 2011 [37]
Pathogen related protein-1	*PR-1*	TTCGGGTTGGATGTGCTAAG	GGTTGAAGCTCAATGGCACT	Medtr2g435490	This work
Protease inhibitor	*PI*	TGTGGTGCAATTCTTTCAGG	ATTTTGGGGTGAGGTGTTGA	Medtr4g032865	This work
Leghemoglobin-1	*MtLb1*	ATAGCTCATATGAGGCATTCAA	GAGTTGAGGACTATCTTGTACT	Medtr5g066070	Li X et al., 2018 [42]
Cysteine proteinase	*Cp6*	CCTGCTGCTACTATTGCTGGATATG	CACTCGCATCAATGGCTACGG	Medtr4g079800	Pierre et al., 2014 [43]

Tables S2 to S10: Statistical results for each experiment.

Table S2. Aphid survival on SNF and NI plants. Results of Two-way ANOVA analysis showing the absence of significant effect of the plant condition (SNF and NI) on the aphid survival $F (1, 170) = 0.2217$, $P = 0.6384$. There were significant differences among the different aphid lines $F (4, 170) = 5.338$, $P = 0.0004$.

Aphid lines	Signifiance	p value
SNF Ri(n) vs. SNF Hd	ns	0.9992
SNF Ri(n) vs. SNF Ss	ns	0.3572
SNF Ri(n) vs. SNF Amp	ns	0.9992
SNF Ri(n) vs. SNF. Ri(a)	ns	0.1399
SNF Ri(n) vs. NI Ri(n)	ns	0.9992
SNF Ri(n) vs. NI Hd	ns	0.9744
SNF Ri(n) vs. NI Ss	ns	0.8999
SNF Ri(n) vs. NI Amp	ns	>0.9999
SNF Ri(n) vs. NI Ri(a)	ns	0.3572
SNF Hd vs. SNF Ss	ns	0.8351
SNF Hd vs. SNF Amp	ns	0.8999
SNF Hd vs. SNF Ri(a)	ns	0.5444
SNF Hd vs. NI Ri(n)	ns	>0.9999
SNF. Hd vs. NI Hd	ns	>0.9999
SNF Hd vs. NI Ss	ns	0.9992
SNF. Hd vs. NI Amp	ns	>0.9999
SNF. Hd vs. NI Ri(a)	ns	0.8351
SNF. Ss vs. SNF Amp	ns	0.0685
SNF Ss vs. SNF Ri(a)	ns	>0.9999
SNF Ss vs. NI Ri(n)	ns	0.8351
SNF. Ss vs. NI Hd	ns	0.9744
SNF. Ss vs. NI Ss	ns	0.9967
SNF. Ss vs. NI Amp	ns	0.6571
SNF Ss vs. NI Ri(a)	ns	>0.9999
SNF Amp vs. SNF Ri(a)	*	0.0175
SNF Amp vs. NI Ri(n)	ns	0.8999
SNF Amp vs. NI Hd	ns	0.6571
SNF Amp vs. NI Ss	ns	0.4527
SNF Amp vs. NI Amp	ns	0.9744
SNF Amp vs. NI Ri(a)	ns	0.0685
SNF Ri(a) vs. NI Ri(n)	ns	0.5444
SNF Ri(a) vs. NI Hd	ns	0.8276
SNF Ri(a) vs. NI Ss	ns	0.942
SNF Ri(a) vs. NI Amp	ns	0.3482
SNF Ri(a) vs. NI Ri(a)	ns	>0.9999
NI Ri(n) vs. NI Hd	ns	>0.9999
NI Ri(n) vs. NI Ss	ns	0.9992

NI Ri(n) vs. NI Amp	ns	>0.9999
NI Ri(n) vs. NI Ri(a)	ns	0.8351
NI Hd vs. NI Ss	ns	>0.9999
NI Hd vs. NI Amp	ns	0.9992
NI Hd vs. NI Ri(a)	ns	0.9744
NI Ss vs. NI Amp	ns	0.9898
NI Ss vs. NI Ri(a)	ns	0.9967
NI Amp vs. NI Ri(a)	ns	0.6571

Table S3. Weight of aphids on SNF and NI plants. Results of Two-way ANOVA analysis showing a significant effect of the plant condition (SNF and NI), F (1, 170) = 209.2, P < 0.0001 and of the aphid line, F (4, 170) = 9.073, P < 0.0001, on the aphid weight. There was no significant interaction between the "aphid line" and the weight of aphids F (4, 170) = 1.763, P=0.1386.

Aphid lines	Significance	p value
SNF Ri(n) vs. SNF Hd	ns	0.5642
SNF Ri(n) vs. SNF Ss	ns	>0.9999
SNF Ri(n) vs. SNF Amp	ns	0.226
SNF Ri(n) vs. SNF. Ri(a)	ns	>0.9999
SNF Ri(n) vs. NI Ri(n)	****	<0.0001
SNF Ri(n) vs. NI Hd	****	<0.0001
SNF Ri(n) vs. NI Ss	****	<0.0001
SNF Ri(n) vs. NI Amp	****	<0.0001
SNF Ri(n) vs. NI Ri(a)	****	<0.0001
SNF Hd vs. SNF Ss	ns	0.7689
SNF Hd vs. SNF Amp	ns	>0.9999
SNF Hd vs. SNF Ri(a)	ns	0.8633
SNF Hd vs. NI Ri(n)	****	<0.0001
SNF. Hd vs. NI Hd	****	<0.0001
SNF Hd vs. NI Ss	****	<0.0001
SNF. Hd vs. NI Amp	****	<0.0001
SNF. Hd vs. NI Ri(a)	ns	0.0743
SNF. Ss vs. SNF Amp	ns	0.3996
SNF Ss vs. SNF Ri(a)	ns	>0.9999
SNF Ss vs. NI Ri(n)	****	<0.0001
SNF. Ss vs. NI Hd	****	<0.0001
SNF. Ss vs. NI Ss	****	<0.0001
SNF. Ss vs. NI Amp	****	<0.0001
SNF Ss vs. NI Ri(a)	***	0.0002
SNF Amp vs. SNF Ri(a)	ns	0.521
SNF Amp vs. NI Ri(n)	***	0.0006
SNF Amp vs. NI Hd	****	<0.0001
SNF Amp vs. NI Ss	****	<0.0001
SNF Amp vs. NI Amp	****	<0.0001

SNF Amp vs. NI Ri(a)	ns	0.2675
SNF Ri(a) vs. NI Ri(n)	****	<0.0001
SNF Ri(a) vs. NI Hd	****	<0.0001
SNF Ri(a) vs. NI Ss	****	<0.0001
SNF Ri(a) vs. NI Amp	****	<0.0001
SNF Ri(a) vs. NI Ri(a)	***	0.0003
NI Ri(n) vs. NI Hd	ns	0.5809
NI Ri(n) vs. NI Ss	ns	0.997
NI Ri(n) vs. NI Amp	**	0.0037
NI Ri(n) vs. NI Ri(a)	ns	0.6331
NI Hd vs. NI Ss	ns	0.9778
NI Hd vs. NI Amp	ns	0.617
NI Hd vs. NI Ri(a)	**	0.0041
NI Ss vs. NI Amp	ns	0.0626
NI Ss vs. NI Ri(a)	ns	0.1371
NI Amp vs. NI Ri(a)	****	<0.0001

Table S4. Dry weight of SNF plants. Results of the Tukey multiple-comparison test analysing the effect of the different pea aphid lines (YR2 genotype) on the dry weight of SNF plants; One-way ANOVA of the whole experiment, $F (5, 102) = 5.989$, $P<0.0001$.

Conditions	Signifiance	p value
Control vs. Amp	***	0.0002
Control vs. Ri(n)	ns	0.1022
Control vs. Ri(a)	*	0.0152
Control vs. Hd	ns	0.4733
Control vs. Ss	***	0.0003
Amp vs. Ri(n)	ns	0.4011
Amp vs. Ri(a)	ns	0.8334
Amp vs. Hd	ns	0.0772
Amp vs. Ss	ns	>0.9999
Ri(n) vs. Ri(a)	ns	0.9808
Ri(n) vs. Hd	ns	0.9627
Ri(n) vs. Ss	ns	0.4315
Ri(a) vs. Hd	ns	0.6463
Ri(a) vs. Ss	ns	0.8567
Hd vs. Ss	ns	0.0872

Table S5. Dry weight of NI plants. Results of the Tukey multiple-comparison test analysing the effect of the different pea aphid lines (YR2 genotype) on the dry weight of NI plants; One-way ANOVA of the whole experience, F (5, 102) = 4.035, P = 0.0022.

Conditions	Signifiance	p value
Control vs. Amp	*	0.039
Control vs. Ri(n)	**	0.0046
Control vs. Ri(a)	**	0.0018
Control vs. Hd	*	0.0409
Control vs. Ss	ns	0.0847
Amp vs. Ri(n)	ns	0.9812
Amp vs. Ri(a)	ns	0.9227
Amp vs. Hd	ns	>0.9999
Amp vs. Ss	ns	0.9997
Ri(n) vs. Ri(a)	ns	0.9998
Ri(n) vs. Hd	ns	0.9791
Ri(n) vs. Ss	ns	0.9159
Ri(a) vs. Hd	ns	0.9173
Ri(a) vs. Ss	ns	0.7942
Hd vs. Ss	ns	0.9997

Table S6. Number of nodules / SNF plants. Results of the Tukey multiple-comparison test analysing the effect of the different pea aphid lines (YR2 genotype) on the number of nodules per plant for SNF plants; One-way ANOVA of whole experiment, F (5, 30) = 14.54, P < 0.0001.

Conditions	Signifiance	p value
Control vs. Amp	****	<0.0001
Control vs. Ri(n)	*	0.0156
Control vs. Ri(a)	***	0.0002
Control vs. Hd	ns	0.2316
Control vs. Ss	****	<0.0001
Amp vs. Ri(n)	ns	0.0755
Amp vs. Ri(a)	ns	0.7837
Amp vs. Hd	**	0.0036
Amp vs. Ss	ns	0.9593
Ri(n) vs. Ri(a)	ns	0.6313
Ri(n) vs. Hd	ns	0.8107
Ri(n) vs. Ss	*	0.0105
Ri(a) vs. Hd	ns	0.0844
Ri(a) vs. Ss	ns	0.3003
Hd vs. Ss	***	0.0004

Table S7. Weight of nodules per plant. Results of the Tukey multiple-comparison test analyzing the effect of the different pea aphid lines (YR2 genotype) on the weight of nodules in SNF plants (mg / plant); One-way ANOVA of whole experiment, $F (5, 30) = 7.195$, $P < 0.0002$.

Conditions	Significance	p value
Control vs. Amp	***	0.0003
Control vs. Ri(n)	ns	0.552
Control vs. Ri(a)	ns	0.1356
Control vs. Hd	ns	0.419
Control vs. Ss	***	0.0008
Amp vs. Ri(n)	*	0.0284
Amp vs. Ri(a)	ns	0.1895
Amp vs. Hd	*	0.0474
Amp vs. Ss	ns	0.9997
Ri(n) vs. Ri(a)	ns	0.9464
Ri(n) vs. Hd	ns	>0.9999
Ri(n) vs. Ss	ns	0.0563
Ri(a) vs. Hd	ns	0.9841
Ri(a) vs. Ss	ns	0.314
Hd vs. Ss	ns	0.0909

Table S8. ARA per mg of nodules. Results of the Tukey multiple-comparison test analysing the effect of the different pea aphid lines (YR2 genotype) on ARA per mg of nodules in SNF plants; One-way ANOVA of whole experiment, $F (5, 30) = 5.285$, $P < 0.0013$.

Conditions	Significance	p value
Control vs. Amp	*	0.0118
Control vs. Ri(n)	ns	0.3023
Control vs. Ri(a)	ns	0.0811
Control vs. Hd	*	0.0289
Control vs. Ss	***	0.0005
Amp vs. Ri(n)	ns	0.6566
Amp vs. Ri(a)	ns	0.9617
Amp vs. Hd	ns	0.9991
Amp vs. Ss	ns	0.8543
Ri(n) vs. Ri(a)	ns	0.9808
Ri(n) vs. Hd	ns	0.8551
Ri(n) vs. Ss	ns	0.1113
Ri(a) vs. Hd	ns	0.9973
Hd vs. Ss	ns	0.6556

Table S9. ARA per plant. Results of the Tukey multiple-comparison test analysing the effect of the different pea aphid lines (YR2 genotype) on ARA per plant; One-way ANOVA of whole experiment, F (5, 30) = 15.15, P < 0.0001.

Conditions	Signifiance	p value
Control vs. Amp	****	<0.0001
Control vs. Ri(n)	ns	0.1004
Control vs. Ri(a)	**	0.0014
Control vs. Hd	**	0.0062
Control vs. Ss	****	<0.0001
Amp vs. Ri(n)	**	0.0017
Amp vs. Ri(a)	ns	0.1154
Amp vs. Hd	*	0.0333
Amp vs. Ss	ns	>0.9999
Ri(n) vs. Ri(a)	ns	0.5216
Ri(n) vs. Hd	ns	0.8474
Ri(n) vs. Ss	**	0.0011
Ri(a) vs. Hd	ns	0.9927
Hd vs. Ss	*	0.0225

Table S10. t-test analysis of the expression of *PR1, PI, Mtbl1* and *MtCp6* genes. Comparison of plants infested by the different aphid lines with non-infested controls. Significant values are highlighted in yellow.

	p value	p value
	MtLb-1	***MtCp6***
**Amp SNF**	0.436704	0.00961
**Ri(n) SNF**	0.055844	0.147555
**Ri(a) SNF**	0.041158	0.00127
**Hd SNF**	0.034613	0.024828
**Ss SNF**	0.128093	0.000791

	p value	p value	p value	p value
	***PR1* NI**	***PR1* SNF**	***PI* NI**	***PI* SNF**
**Amp**	0.005208	0.015178	0.108835	0.000478
**Ri(n)**	0.002926	0.00413	0.457504	0.00073
**Ri(a)**	0.000892	0.011606	0.132567	0.001503
**Hd**	0.099349	0.00384	0.525784	9.92E-05
**Ss**	0.004047	0.01034	0.243729	0.000885

Table S11. Raw data and SEM for figure 3.

	Figure 3 A		Figure 3 B		Figure 3 C		Figure 3 D	
**Sample**	**Mean**	**SEM**	**Mean**	**SEM**	**Mean**	**SEM**	**Mean**	**SEM**
**Control**	15.84	± 0.55	3.75	± 0.18	14.2	± 0.29	52.40	± 2.16
**Amp**	9.01	± 0.86	2.02	± 0.19	12.31	± 0.25	24.75	± 2.56
**Ri(n)**	12.05	± 0.34	3.16	± 0.13	13.1	± 0.17	41.82	± 1.06
**Ri(a)**	10.38	± 1.23	2.85	± 0.28	12.73	± 0.34	35.08	± 3.01
**Hd**	13.38	± 0.29	3.08	± 0.15	12.5	± 0.58	37.27	± 1.34
**Ss**	8.11	± 0.80	2.12	± 0.41	11.71	± 0.40	24.12	± 4.70

	Figure 3 E		Figure 3 F	
**Sample**	**Rescaled expression**	**SEM**	**Rescaled expression**	**SEM**
**Control**	1	± 0.29	1	± 0.46
**Amp**	0.74	± 0.41	12.23	± 6.39
**Ri(n)**	0.23	± 0.09	6.90	± 2.38
**Ri(a)**	0.37	± 0.06	23.28	± 10.13
**Hd**	0.34	± 0.06	29.34	± 15.19
**Ss**	0.21	± 0.09	11.36	± 1.69
